# Supplementary material for: Estimates of Dietary Exposure to Antibiotics among a Community Population in East China
Source: Antibiotics (Basel). 2022 Mar 17;11(3):407. doi: 10.3390/antibiotics11030407 (PMC8944873; doi:10.3390/antibiotics11030407)
Supplement: Supplementary file 1 [file antibiotics-11-00407-s001.zip › antibiotics-1620202-supplementary.pdf]

Supplementary materials

Estimates of dietary exposure to antibiotics among a community population in East China

Yingying Wang [1,†], Xinping Zhao [1,†], Jinxin Zang [1,†], Yurong Li [1], Xiaolian Dong [2], Feng Jiang [1], Na Wang [1#], Lufang Jiang [1], Qingwu Jiang [1] and Chaowei Fu [1#]

[1].School of Public Health; Key Laboratory of Public Health Safety; NHC Key Laboratory of Health Technology Assessment, Fudan University, Shanghai, 200032, China;

[2].Deqing County Center for Disease Prevention and Control, Huzhou, 550004, China.

† These authors contributed equally: Yingying Wang, Xinping Zhao, Jinxin Zang.

* Correspondence: Na Wang, E-mail: na.wang@fudan.edu.cn; Chaowei Fu, E-mail:fcw@fudan.edu.cn.

Table S1 Detection concentrations of selected twenty-one antibiotics in animal-derived foods from the notification of unqualified edible agricultural products after special supervision sampling inspection in Deqing County

Antibiotics	Usage [a]	Food categories and detection concentrations
Tetracyclines		
Tetracycline	H/VA	prawn (553 μg/kg)
Oxytetracycline	H/VA	beef (320 μg/kg), chicken (342 μg/kg), prawn (289 μg/kg), prawn (497 μg/kg), prawn (379 μg/kg), prawn (440 μg/kg), prawn (102 μg/kg)
Chlortetracycline	VA	-
Fluoroquinolones		
Ciprofloxacin	H/VA	-
Ofloxacin	H/VA	snakehead (42.8 μg/kg), snakehead (8.55 μg/kg), large yellow croaker (21.7 μg/kg), small yellow croaker (68.4 μg/kg), bull frog (35.8 μg/kg)
Norfloxacin	H/VA	-
Enrofloxacin	VA	crucian (1400 μg/kg), crucian (267 μg/kg),weever (1600 μg/kg), butterfish (240 μg/kg), butterfish (1600 μg/kg), prawn (498 μg/kg), prawn (250 μg/kg), swamp ell (2636.4 μg/kg), bull frog (655.8 μg/kg), bull frog (577 μg/kg), bull frog (270 μg/kg)
Macrolides		
Azithromycin	HA	-
Roxithromycin	HA	-
Clarithromycin	HA	-
Erythromycin	H/VA	-
Tilmicosin	VA	-
Sulfonamides		
Trimethoprim	H/VA	chicken (288 μg/kg), chicken (69.0 μg/kg), snakehead (82.7μg/kg)
Sulfadiazine	H/VA	-
Sulfamethoxazole	H/VA	-
Sulfamethazine	H/VA	pork (136 μg/kg), chicken (907 μg/kg), chicken (819 μg/kg), large yellow croaker (145 μg/kg), prawn (457μg/kg), chicken egg (64.6 μg/kg)

Acetylated sulfamethoxazole	H/VA	-
Acetylated sulfamethazine	H/VA	-
Phenicols		
Chloramphenicol	HA	beef (0.52 μg/kg), gizzard (1.07 μg/kg), sausage (0.8 μg/kg), crucian (4.4 μg/kg), clam (4.5 μg/kg), clam (11.4 μg/kg)
Thiamphenicol	H/VA	duck egg (0.2μg/kg), quail egg (0.5 μg/kg)
Florfenicol	VA	chicken egg (5.3 μg/kg), chicken egg (78.9 μg/kg), duck egg (8.88 μg/kg), duck egg (13.5μg/kg), duck egg (48.6μg/kg),

[a] Usage: VA, veterinary antibiotic exclusively used in animal; HA, human antibiotic exclusively used in human; H/VA, human/veterinary antibiotic used in both animal and human.
